# Perceptions and experiences of Hospital-Acquired Diarrhea among children aged 0–59 months with Severe Acute Malnutrition at Mwanamugimu Nutrition Unit, Mulago, Uganda: A qualitative study

**DOI:** 10.1371/journal.pone.0340572

**Published:** 2026-01-06

**Authors:** Mahad Muweesi, Agnes Nyabigambo, Arthur Bagonza, Musaazi John Francis Kimuli, Grace Rickson Nsubuga, Christopher Garimoi Orach

**Affiliations:** Department of Community Health and Behavioural Sciences, School of Public Health, Makerere University College of Health Sciences, Kampala, Uganda; Cranfield University, UNITED KINGDOM OF GREAT BRITAIN AND NORTHERN IRELAND

## Abstract

**Background:**

Hospital-Acquired Diarrhea (HAD) is a global health threat and a leading cause of illness and death in children with Severe Acute Malnutrition (SAM). In Uganda, HAD significantly affects children under five with SAM, creating a heavy health and economic burden. This study explored caregivers’ and health workers’ perceptions and experiences of HAD among children aged (0–59) months with SAM regarding HAD at Mwanamugimu Nutrition Unit, Mulago, Uganda.

**Methods:**

A qualitative study was conducted at Mwanamugimu Nutrition Unit, involving 12 caregivers and 10 health workers. Audio-recorded data were collected through in-depth interviews (IDIs) and key informant interviews (KIIs). Data was managed using Atlas ti version 6 and thematically analyzed to identify perceptions and experiences of HAD among children (0–59) months with SAM at Mwanamugimu.

**Results:**

The results are presented according to the major themes that emerged from the data: understanding health risk of HAD and their impact, advantages and challenges of health interventions, empowerment through health education, prompts and motivators for action. Caregivers reported emotional distress, inadequate communication with patient progress by health workers, limited family social support, difficulty in maintaining clean toilets, and financial constraints for diapers and other sanitary items as contributors to HAD. Health workers attributed HAD to inconsistent water supply, inadequate dietary knowledge, and poor infection control. Both groups stressed that motivational triggers are essential for sustainable adherence to management guidelines.

**Conclusion:**

This study emphasizes the urgent need to empower caregivers through targeted health education, adequate hygiene support, and structured ward routines. Strengthening caregiver confidence and participation enhances adherence and recovery among children with severe acute malnutrition. Integrating psychosocial support and participatory strategies into hospital nutrition programs can reduce hospital-acquired diarrhea and promote sustainable, high-quality child health outcomes.

## Background

Approximately 1.6 million deaths occur each year globally due to diarrhea, with the highest burden in developing countries and economically disadvantaged regions [1,2]. Hospital-Acquired Diarrhea (HAD) is a significant concern in pediatric healthcare settings worldwide. In the United States, around 20,000 cases of *Clostridioides difficile* infection (CDI), a leading cause of HAD, are reported annually among children [3]. In low- and middle-income countries (LMICs), particularly in sub-Saharan Africa, the burden is even more pronounced due to limited resources and healthcare infrastructure. In sub-Saharan Africa, Uganda stands out with the highest mortality rate for children under five, where 0–22% of these deaths are attributed to HAD [4].

Hospital-acquired diarrhea (HAD) refers to diarrhea contracted from a health care setting where patients admitted for different medical conditions develop watery stool after 72 hours of admission or 48 hours post discharge from the hospital [5]. In the context of severe acute malnutrition (SAM) treatment, this condition is of particular concern due to the susceptibility of children with compromised nutritional status leading to dehydration, prolonged hospital stays, increased healthcare costs, electrolyte imbalances, organ dysfunction, and compromised nutritional recovery in malnourished children [6]. Hospitalization often exposes these children to various pathogens and risk factors that can trigger or exacerbate diarrhea [7]. Severe acute malnutrition (SAM) poses serious health risk of HAD, as malnourished children have weakened immunity, making them highly susceptible to infections [8]. HAD exacerbates dehydration, electrolyte imbalances, and nutrient loss, delaying recovery and increasing mortality hence the vicious cycle [9].

Most cases of diarrhea in hospitalized patients are caused by *Clostridium difficile* [10]. Laboratory guidelines for hospital-acquired diarrhea employ culture-based methods for bacterial identification, including *Salmonella, Shigella*, and *Campylobacter* utilize PCR for detecting *Clostridium difficile* and norovirus test for parasites like *Giardia* and *Cryptosporidium* using microscopy or antigen detection [11].

The evaluation of a hospitalized patient in whom diarrhea develops should initially focus on the clinical presentation and attention to signs of sepsis. Stable patients with mild symptoms may respond to withdrawal of the offending agent (if any), while patients with moderate or severe symptoms including those with fever, hypotension, leukocytosis, acute kidney injury, or a decreased serum bicarbonate level should be tested for *C. difficile* infection [12].

Healthcare providers, including doctors, nurses, and nutritionists, are responsible for diagnosing and treating diarrhea in these children [13]. They follow established treatment guidelines, which include the provision of oral rehydration solutions (ORS), dietary adjustments for those on normal food rations, and ready to use therapeutic feeds (F75, F100 and RUTF) to manage the condition effectively [14].

A study in Bangladesh that involved three tertiary public hospitals highlighted that less rigorous infection control measures increase the risk of acquiring pathogens during hospital stays [15]. In contrast, a study done in Uganda highlights that Uganda’s healthcare system is plagued by poor infrastructure, including a lack of clean water, inadequate housing for healthcare staff, and insufficient medical equipment all of which can facilitate the spread of infections within healthcare facilities [16].

The economic burden of diarrhea extends beyond healthcare expenditures, affecting families’ productivity and potentially perpetuating the cycle of poverty [17]. As such, mitigating the risk and impact of diarrhea is a critical public health goal, especially among vulnerable populations like children with acute malnutrition [18].

Addressing HAD requires both treatment and prevention. Key treatment measures usually prescribed for caregivers with children experiencing HAD include; oral rehydration therapy, zinc supplementation, and therapeutic feeds for managing diarrhea [19]. Relatedly, healthcare workers are expected to ensure strict hand hygiene and proper sterilization of medical equipment in response to the expected prevention measures [20]. However, HAD’s complex causes call for further research to develop targeted, evidence-based strategies in healthcare settings [21].

Despite these efforts, it remains unclear how caregivers perceive, recognize, organize and interpret information about HAD, and whether they understand the role the environment plays in its acquisition. Moreover, uncertainty exists regarding whether healthcare workers possess adequate practical knowledge and skills typically gained through direct involvement in mitigation activities to address HAD effectively. This study therefore explored health workers’ and caregivers’ perceptions and experiences regarding HAD among children aged 0–59 months with SAM at Mwanamugimu Nutrition Unit, Mulago, Uganda.

## Methods

This study adopted a qualitative descriptive design aimed at exploring the perceptions and experiences of healthcare workers and caregivers regarding hospital-acquired diarrhea (HAD) among children aged 0–59 months with severe acute malnutrition (SAM) at Mwanamugimu Nutrition Unit, Mulago National Referral Hospital, Uganda. The methodology followed the Consolidated Criteria for Reporting Qualitative Studies (COREQ) checklist to ensure rigor and transparency [22].

### Study design and approach

This study was a qualitative descriptive observational study that employed purposive sampling to recruit healthcare workers and caregivers of children with SAM. In-depth interviews (IDIs) and key informant interviews (KIIs) were conducted to onbtain rich, contextual insights. This approach was chosen to provide a detailed understanding of participants’ perceptions and experiences regarding HAD in a real-world hospital setting. The design emphasized participants’ lived experiences and practical knowledge, consistent with Sandelowski’s recommendation for qualitative descriptive research [23].

### Study participants and sampling

The study population consisted of caregivers of children 0–59 months with SAM and healthcare providers involved in their care. Purposive sampling was used to ensure inclusion of participants with rich, relevant experiences. A total of 22 interviews were conducted: 12 IDIs with biological mothers of children admitted with SAM and 10 KIIs with healthcare workers (1 pediatrician, 3 medical officers, 4 nutritionists, and 2 nurses). Among the key informants, seven were female and three were male, mostly aged between 25 and 35 years. All IDI participants were mothers; nine were aged 15–24 years, and three were 25 years or older. Seven were married, while five were unmarried. Participants were recruited until data saturation was reached, when no new information emerged in subsequent interviews. Caregivers of children with congenital anomalies or deteriorating conditions and healthcare workers unwilling to participate were excluded.

### Data collection procedures

Data were collected over a three-month period, from June 1 to August 31, 2024, using in-depth interviews (IDIs) and key informant interviews (KIIs). The interview guides were developed by the research team in English and reviewed by two qualitative research experts for content validity. The guides were then translated into *Luganda* by the entire research team in one of the meeting held at the unit to ensure clarity and cultural appropriateness. A pilot study was conducted from May 23 to May 30, 2024, at Mwanamugimu Nutrition Unit to assess the clarity, flow, and contextual relevance of the interview guides before full-scale data collection.

Since the research assistants were nutritionists already working at the Mwanamugimu Nutrition Unit, they played a key role in identifying eligible participants. Each day, they reviewed patient admissions and requested information from colleagues about children who developed diarrhea during hospitalization. Caregivers of these children were then approached and invited to participate in the study. The principal researcher visited the unit on alternating days to oversee the recruitment and data collection process. Additionally, an announcement was made every morning during ward rounds, informing all staff that any child who developed diarrhea should be reported to the research team for potential inclusion in the study. Because all healthcare staff were aware of the ongoing study, cooperation in participants identification and referral was smooth and systematic.

The caregiver interviews explored perceptions and experiences if diarrhea onset during hospitalization, hygiene practices, communication with health workers, and the perceived effects of hospital-acquired diarrhea. The healthcare worker interviews focused on infection prevention and control practices, diagnostic procedures, and institutional challenges. The interview guides were developed with reference to relevant literature on the healthcare-associated infections and hospital-acquired diarrhea [24].

Key informant interviews were conducted in English, while in-depth interviews were mostly conducted in *Luganda*. Research assistants fluent in both English and *Luganda* were trained and deployed to facilitate effective communication and accurate interpretation during interviews. The researcher and his team were officially introduced to the healthcare staff and caregivers at Mwanamugimu Nutrition Unit by the Head of Department before the commencement of data collection to build trust and promote cooperation.

All interviews were conducted face-to-face in quiet spaces within or near the ward to ensure privacy and minimize disruptions. Each session was audio-recorded using a decoder and timed with a watch to last between 30 and 60 minutes. The principal investigator, a trained qualitative researcher with a background in public health nutrition, led the interviews with support from a research assistant.

Interviews were conducted in a busy hospital environment, and the research team faced challenges such as ward congestion and limited private space. To minimize interruptions, sessions were scheduled at convenient times for both caregivers and staff, ensuring comfort and the collection of rich, detailed information.

### Research team, reflexivity, quality control, and quality assurance

Data collection at Mwanamugimu Nutrition Unit was conducted by three researchers with expertise in nutrition and qualitative research. The principal researcher (MM), a master’s student, conducted six KIIs and six IDIs, while the remaining four KIIs and six IDIs were conducted by two research assistants (MM and JN), both nutritionists at the unit. Nutritionists were selected based on their experience identifying and recruiting diarrhea cases among children, ensuring data richness and accuracy. The research assistants existing rapport with participants facilitated openness during interviews, and the purpose of the study, researcher affiliations, and assurances of confidentiality were explained before data collection. To ensure quality and consistency, research assistants underwent two days of refresher training covering study objectives, ethical conduct, interview techniques, and use of data collection tools, with ongoing supervision and feedback sessions during data collection. Pilot testing of the IDI and KII guides with caregivers and healthcare workers assessed clarity, cultural appropriateness, and flow, with minor adjustments made to improve comprehension and relevance. All interviews were audio-recorded, English recordings were transcribed verbatim, and *Luganda* recordings were translated by the research team. The principal investigator reviewed all transcripts against recordings to ensure fidelity.

The team acknowledged that their professional backgrounds as nutritionists and healthcare providers could influence interpretation, particularly regarding biomedical explanations of diarrhea and malnutrition. Ongoing reflexive discussions were conducted to examine positionality and assumptions, ensuring participants’ perspectives were prioritized during coding and analysis.

### Data management and analysis

The researchers employed a thematic analysis model using both deductive and inductive approaches. To ensure a deep understanding of the data, transcripts were repeatedly read through to become familiar with the content. The researchers independently reviewed the transcripts and developed initial codes. Coding disagreements were discussed in joint meetings, and consensus was reached through deliberation until all three researchers agreed on the final codebook. This process enhanced reliability and reduced individual bias in coding. Consensus of the developed codes was reached which was then used to code the remaining transcripts. The codes captured specific concepts, ideas, or phenomena related to caregivers’ and healthcare workers’ experiences and perceptions of hospital-acquired diarrhea (HAD). Data analysis was performed using thematic analysis in ATLAS.ti 6 software to systematically organize and interpret the findings. Credibility and trustworthiness were ensured through triangulation of caregiver and health worker perspectives, regular team debriefings during coding, and inclusion of verbatim quotations to support key themes.

All transcripts were de-identified before analysis and inclusion as supplementary materials. Identifying information such as names, specific locations, and personal identifiers were replaced with neutral codes. Each participant was assigned a unique identifier (e.g., C01-C12 for caregivers and K01-K10 for health workers). The de-identification ensured participant confidentiality while maintaining the authenticity of the data. The de-identified transcripts are provided as supplementary files accompanying this manuscript.

### Ethics approval and consent to participate

Ethical approval for this study was obtained from the Makerere University School of Public Health Higher Degrees, Research and Ethics Committee (HDREC) (MakSPH-REC 2024/416). Administrative clearance was also granted by the Mulago National Referral Hospital (MH-REC 2024/2756). Written informed consent was obtained from caregivers of all participating children before the commencement of interviews. For caregivers who could not read or write, consent was obtained through thumbprints after a detailed explanation in their preferred language. To safeguard participant confidentiality, each caregiver was assigned a unique identification number instead of names on transcripts and a data files. Only the principal investigator had access to the link between participant codes and identifies, which was destroyed after transcription.

## Results

The qualitative findings are presented into four main themes: understanding health risk of HAD and their impact, advantages and challenges of health interventions, empowerment through health education, prompts and motivators for action. All 22 eligible participants, including 10 key informants and 12 in-depth interviews, were enrolled in the study, giving 100% response rate (**Table 1**).

**Table 1:** Coding tree illustrating themes and subthemes on caregivers’ perception and experiences of hospital-acquired diarrhea (HAD) among children among children with Severe Acute Nutrition (SAM).

S/NO	Theme	Sub-theme	Code	Description	Quote
**1**	Health risk of HAD & their impact	Risk	Concern/unconcerned	Immediate threats like dehydration, death	“he may die”
		Complication	Dreadful	Long-term effects like stunting	“lost weight”
**2**	Advantages and challenges of health interventions	Benefit	Management	Life-saving treatment (feeds, Resomal)	“helped the progress
		Hindrance	Costs	Barriers from expenses (soap, drugs)	“lack financial support”
**3**	Empowerment via health education	Confidence	Health education	Knowledge builds caregiver skills	“I’m in position to implement”
**4**	Prompts and motivators for action	Triggers	Communication channels	Reminders through bells, charts	“respond to the bells”

### Understanding health risk of HAD and their impact

This theme revealed that hospital-acquired diarrhea (HAD) increased health risks for children, including dehydration, malnutrition, abdominal pain, electrolyte imbalances, and delayed recovery from illness such as pneumonia, sickle cell anemia, TB, or HIV/AIDS. HAD also placed heavy emotional and financial burdens on caregivers, these caregivers reported stress, helplessness, prolonged hospital stays, and extra medical costs. Limited communication from healthcare workers worsened caregiver anxiety, highlighting how HAD affects both child health and family well-being.

*“My baby has lost appetite for food, the body has generally become weak, and has also lost weight, and we are even scared that if not managed well he may die; this is worsened by the fact that most times doctors have been checking in my child’s file, measuring the body weight and temperature, pulse and simply writing in our file and leaving without explaining my child’s progress.”* **Participant 03, 27 years**

Most of the key informants revealed the vicious relationship between diarrhea and malnutrition as malnutrition predisposes children to diarrhea and vice versa. They acknowledged that prolonged diarrhea could delay childhood milestones such as walking, talking and cognitive development.

*“Diarrhea causes depletion of vital micronutrients and vitamins which cause malnutrition, malnutrition, in turn, worsens diarrhea through weakening immunity and damaging gut lining increasing susceptibility to infections like diarrhea. Deficiencies in zinc and vitamin A impair intestinal function, leading to poor absorption, creating a vicious cycle of illness. We emphasized the role of breast milk on brain development and improving cognitive abilities up to adulthood”* **Health worker 2, 37 years.**

### Advantages and challenges of health interventions

This theme delved into the advantages of health interventions as well as the challenges faced, which contributed to improving children’s health and recovery. Both formal interventions, such as therapeutic feeding, hygiene demostrattions, and childcare training, and informal recommendations from health workers during ward rounds supported caregivers in applying practices effectively. Together, these approaches provided a structured and reinforced system that enhanced children’s recovery.

Caregivers acknowledged the benefits of following recommended practices, including proper use of therapeutic feeds, good hygiene, hand washing, safe storage of feeding cups, and regular diaper changes. However, financial constraints, limited facilities, and the emotional strain of caring for the sick child often hindered implementation.

*“I lost my job and I’m not working and I have remained jobless until this time, plus I lack sufficient social and financial support from my husband”.* **Participant 07, 34 years**

Most caregivers in the IDIs found the health workers’ recommendations during ward rounds to be highly beneficial not only for their children’s immediate well-being but also as lifelong skills. Additionally, the unit’s guidelines provided training in standard practices for proper child feeding, hygiene, and sanitation, further reinforcing these lessons.

*“I greatly believe these recommendations have very much helped the progress of my child’s healing process, good hygiene is key to preventing frequenting the hospital due to illness-related poor hygiene even before your next appointment arrives, so following recommendations is very paramount according to my experience”* **Participant 10, 28 years**

However, many caregivers faced challenges like poor sanitation in communal toilets, financial burdens due to prolonged hospital stays and fear of losing their children to diarrhea. Some caregivers also struggled with restrictions on feeding, as their children were restricted on doctor-recommended diets, making it difficult to meet their nutritional needs.

*“I am a leader for Mwanamugimu P.2 but managing good hygiene for toilets being utilized by such a big society is very hard since different people have different ethnicities and perhaps different hygiene disciplines, many times toilets and bathrooms are turning dirty shortly after being cleaned and majority of the mothers complain of contracting urinary tract infections after using them, many of the mothers struggle fulfilling the dietary restrictions and they lack financial support from the spouses to buy soap and diapers and other drugs on a daily basis.”* **Participant 01, 45 years**

A few caregivers mentioned that there were no challenges in managing HAD since the hospital provided most necessities for their children and themselves. Items such as therapeutic feeds, food were supplied by the unit. Additionally, caregivers and visitors received soap and various materials needed during their hospital stay, making the management process easier.

*“I have found no challenges managing this problem because most things which include baby feeds, soap and antiseptics are provided at the hospital at a free cost and I am not so much affected emotionally because my child is improving and I am following the rules as told by the doctors.”* **Participant 02, 27 years**

Most key informants explained the role of the guidelines provided in managing HAD among children with SAM. They highlighted that children with SAM were given therapeutic feeds, zinc, fluids and antibiotics and their progress was monitored through stool charts. Laboratory investigations and hygiene education were conducted to identify and address the causes of diarrhea.

*“We do cross-monitoring with the stool charts to assess if the child still needs Resomal or not, Resomal is prescribed according to the IMAM guidelines where it is given according to body weight only given to the children who have diarrhea number one and two, zinc, fluids and antibiotics are also utilized where necessary, hygiene practices like personal hygiene and environmental sanitation are highly emphasized. We investigate using laboratory tests like CBC and microscopy to establish the cause of the diarrhea and address the causes of HAD appropriately.”* **Health worker 03, 42 years**

A few of the key informants complained that sometimes drugs are prescribed but were not administered on time and understaffed due to the overwhelming number of patients; these affect treatment effectiveness and can lead to worsened conditions. Timely administration is crucial for achieving desired health outcomes and preventing complications. Delays disrupt the treatment regimen, potentially extending recovery time and increasing the risk of adverse effects.

*“As a doctor, some prescriptions I make are not given to the patients as prescribed on time and for the hospital, there’s sometimes understaffing to manage the overwhelming number of patients…”* **Health worker 06, 34 years**

### Empowerment through health education

This theme highlights on the importance of health education in empowering caregivers with the knowledge and confidence to manage HAD. While most caregivers found the recommendations easy to implement, some struggled due to financial constraints and self-doubt. Healthcare workers emphasized the need for structured health education sessions to enhance caregivers’ self-efficacy.

Most caregivers in the IDIs mentioned that the recommendations were easy to implement. They felt empowered by the knowledge provided by the healthcare workers and were motivated to ensure their children’s recovery.

*“I don’t find any hardships implementing the recommendations I’m actually confident that all the health workers want me to do I’m in position to implement with easy because hygiene was already part of daily routine even at home.”* **Participant 02, 27years**

However, some caregivers found the recommendations difficult to follow, particularly those that required finances which they lacked. Their uncertainty in managing their child’s condition reflected low self-efficacy, which could hinder adherence to the guidelines.

*“Some recommendations are really hard for me to implement since some require me to spend money which I did not have.”* **Participant 08, 22 years**

A few key informants emphasized the importance of structured health education sessions to improve caregivers’ confidence and ability to manage HAD. They suggested that evening health talks could provide a calm and interactive platform for educating caregivers on recommended practices, treatment, management and prevention of HAD.

*“Continuous health education during the evening hours could be of a great impact because this is a calm time, educating them about the normal routine of the hospital, protocol, guidelines for disease management and care takers responsibility in disease prevention and health promotion at all stages. Here, we could select a topic about the problem and discuss it freely.”* **Health worker 06, 34 years**

### Prompts and motivators for action

This theme highlights the triggers and encouragements for preventing hospital-acquired diarrhea among children with severe acute malnutrition. Health workers reinforced these through regular health education sessions that equipped caregivers with essential hygiene, feeding, and treatment skills. Visual reminders such as posters, meal bells, and feeding charts served as daily cues that encourage consistent healthy practices. The selection of caregiver leaders promoted peer learning and accountability, while supportive supervision and feedback from nurses and nutritionists built caregivers, confidence and adherence. Observable improvements in child health further motivated caregivers to sustain these behaviors, demonstrating the effectiveness of structure guidance in fostering lasting healthy practices within the pediatric nutrition unit.

Most caregivers emphasized key practices such as hand washing, proper disposal of waste and timely feeding to reduce the risk of HAD. They also highlighted the importance of avoiding bottle feeding, ensuring strict drug administration schedules and responding to meal and medication reminders. Hospital staff particularly nurses and nutritionists play a critical role in educating and motivating caregivers to adopt these best practices.

Most caregivers acknowledged the importance of maintaining proper hygiene and sanitation; they reported that hospital staff regularly emphasized hand washing, timely diaper changes and proper disposal of waste. Furthermore, they were advised to use feeding cups instead of bottles, as the teats of these bottles usually harbor bacteria and increase the risk of diarrhea if not well washed. Adherence to drug administration times and consistent feeding schedules were also encouraged to support recovery.

*“A nutritionist always told us to respond to the bells for serving meals, bring the children every morning for measuring weights and heights and medical assessing during the ward round. She discouraged the use of feeding bottles as they harbor infections and exposes the children to diarrheal diseases, she usually asks us where we keep our feeding cup after washing, and she says poor storage could lead to contracting infections*.” **Participant 09, 42 years**

Most caregivers in the IDIs acknowledged the motivators for action for managing HAD like, selection of patient leaders, routine nutrition status assessment, these facilitated regular weight and height monitoring. They viewed these assessments as essential for tracking their child’s progress and ensuring proper nutrition care. In addition, caregivers appreciated the healthcare workers’ efforts in reassuring them about their children’s recovery which boosted their confidence and adherence to prescribed practices.

*“…milk serving time where even bells were rung to notify the caregivers it was time for a serving, the nutritionists have always asked us to pick the baby’s milk from the kitchen immediately after hearing the bells, they always taken our babies’ weight and height every morning to see if they are progressing and also encouraged us to carry our meal cards to kitchen; meal cards have the type and quantity of milk prescribed by the nutritionist.”* **Participant 05, 25 years**

Most key informants reinforced these hygiene and nutrition practices by ensuring compliance with recommended guidelines. Healthcare workers conducted daily ward rounds to assess patients’ progress, educate caregivers and monitor adherence to feeding and treatment schedules. In addition, reminders such as bells for meals, selecting patient community leaders and providing diarrhea charts have greatly improved patient care and recovery outcomes.

*“We carry out mornings ward rounds every morning to assess patients’ progress and nutritionists also take patients weights, heights, MUAC morning. Every Wednesday from 8.00 am to 2.00 pm we run an Outpatient Therapeutic Clinic where different topics on health are selected to address malnutrition, OTC is the out let point for all children admitted at the unit they are discharged from here. However, there are children from other departments within Mulago hospital who present with moderate acute malnutrition and just visit OTC clinic nutrition education and RUTF feeds on Wednesday then go back to their departments for admission.”* **Health worker 01, 38 years**

## Discussion

This study aimed at exploring the perceptions and experiences from health workers and caregivers about hospital-acquired diarrhea (HAD) among children aged 0–59 months with severe acute malnutrition (SAM) at Mwanamugimu Nutrition Unit. Four themes emerged from the data collected from participants and these were; understanding health risk of HAD and their impact, advantages and challenges of health interventions, empowerment through health education, prompts and motivators for action.

### Understanding health risk of HAD and their impact

The study findings suggest that HAD leads to severe complications including dehydration, gastrointestinal distress, malnutrition, and even mortality. This is similar to observations from a prospect multicenter cohort study conducted at 12 academic and community medical and surgical ICUs in Canada, USA, Poland, and Saudi Arabia which stated that diarrhea can cause complications such as skin damage, dehydration and kidney problems. Kidney related problems however, have not been noticed in the study findings as broadly mentioned in the prospect cohort study above [25]. These adverse outcomes contribute to nutrient loss, weight reduction, and impaired absorption, further compromising immune function, and leading to death. Some of the mentioned outcomes were also appreciated in a Uganda prospective cohort study conducted at Mwanamugimu Nutrition Unit where diarrhea was considered a strong predictor of mortality among children with complicated SAM [26].

Importantly, caregivers not only understood these clinical risks but also experienced significant anxiety regarding their children’s potential exposure to HAD during hospitalization. This anxiety heightened concern and acute awareness of the cyclical relationship between malnutrition and recurrent infections. This is coherent with findings from the community based study conducted in Ethiopia which revealed that there is a cyclic relationship between poor nutrition and repeated episodes of diarrhea [27]. Such insights on anxiety reinforce existing clinical evidence on HAD while emphasizing the emotional and psychological burden on caregivers. Consequently, the results suggest that health systems must address both the medical and emotional needs of families. Integrative interventions that combine effective infection control and nutritional support with targeted health education and improved communication strategies may alleviate caregiver anxiety and enhance overall care for malnourished children.

### Advantages and challenges of health interventions

Existing evidence shows that dietary interventions such as therapeutic and supplementary feeding improve clinical outcomes in children with acute malnutrition by promoting weight gain, recovery, and reduced morbidity [28]. A systematic review has highlighted the efficacy and cost-effectiveness of these interventions in both community and facility-based settings [29]. The current study compliments the earlier findings by incorporating a qualitative perspective, showing that most caregivers reported noticeable improvements in their children’s health such as weight gain and enhanced appetite and viewed the dietary guidance as practical knowledge that fosters better hygiene practices and helps prevent repeated hospital admissions. This caregiver perspective broadens the focus from purely clinical outcomes to include long-term behaviour change and health empowerment. The results support and extend the existing literature by emphasizing the importance of incorporating health education into nutritional interventions to achieve sustainable improvements in child health. Health systems should integrate nutritional interventions with caregiver education to boost both immediate recovery and long-term health. Investing in training that equips caregivers with practical dietary and hygiene knowledge can reduce re-hospitalizations and support sustainable child health. These findings support policies for holistic acute malnutrition management and the allocation of resources for effective communication and caregiver support, ultimately lessening the economic and social burden of malnutrition.

The study findings indicate that most caregivers experienced significant financial burdens during prolonged admissions, incurring costs for sanitary diapers, wipes, soap, supplementary medications, extra bed sheets and clothing. Previous research has also shown that households incur substantial financial burdens when accessing severe acute malnutrition treatment like what the current study found [30,31]. A comparative qualitative analysis study conducted in Ethiopia and Pakistan highlighted that high opportunity costs such as lost income, transportation expenses, and time away from work are major barriers to accessing and remaining in SAM treatment programs [32]. The current study extends these findings by demonstrating that, in addition to the mentioned costs, caregivers face significant out-of-pocket expenses for non-medical supplies. Specifically, many caregivers reported a financial burden from bills for sanitary diapers, wipes, soap, complementary drugs, extra bed sheets, and clothing during prolonged hospital admissions.

While earlier studies emphasize travel costs and lost productivity as major obstacles, the current study findings add a new layer of complexity by revealing hidden costs associated with long inpatient stays. This insight helps explain additional factors that may contribute to HAD and overall economic strain on families, thereby complementing and deepening the existing evidence base. Health systems should include essential non-medical supplies in SAM treatment packages to ease caregivers’ financial burden but also mitigate acquiring HAD in the hospital setting. Subsidies or targeted support can lower out-of-pocket expenses and improve adherence. Policymakers must address these hidden costs in program design, and clear communication about available support services can help caregivers manage expenses and remain engaged.

### Empowerment through health education

The study findings reveal that most caregivers felt confident in implementing the prescribed dietary and hygiene recommendations. They explained that the educational sessions greatly enhanced their understanding of SAM, ultimately boosting their confidence in mitigating HAD. Previous studies have established that caregiver education plays a crucial role in the effective management of SAM similar to the observations in the current study [33]. Educational interventions have been shown to improve caregivers’ understanding of nutrition and hygiene, which is associated with better adherence to treatment protocols and reduced risk of complications such as HAD [34,35]. The current study adds a new dimension by demonstrating that an education session not only enhances caregivers’ knowledge about SAM but also significantly boosts their confidence in implementing prescribed dietary and hygiene recommendations. This increased confidence appears to empower caregivers to take proactive steps in mitigating HAD a phenomenon that extends beyond traditional outcomes reported in other studies. While existing studies, such as the hospital-based research in Bangladesh and the mixed-methods study from Ethiopia, emphasize risk factors and perceived quality of care [35,36], findings of the current study complement these by highlighting the positive impact of targeted health education on caregiver self-efficacy. By linking enhanced understanding to increased confidence in practical management, this study supports and extends the literature on the critical role of caregiver education in improving SAM treatment outcomes and mitigating HAD.

There is a need to integrate structured educational sessions into SAM management to empower caregivers with the knowledge and confidence to implement effective dietary and hygiene practices. This empowerment can lower the incidence of HAD, improve patient outcomes, and reduce healthcare costs. Policymakers should invest in ongoing caregiver training within both community and facility-based programs, enhancing overall care quality, boosting engagement, and reducing re-hospitalization rates and associated economic burdens.

### Prompts and motivators for action

The study findings revealed that both key informants and in-depth interview participants acknowledged that Mwanamugimu’s established structures or hospital protocols effectively promote adherence to prescribed recommendations. These structures or protocols ensure that recommendations are implemented systematically and sustainably, promoting long-term compliance. Studies conducted elsewhere have demonstrated that structured hospital protocols such as checklists can improve adherence to clinical guidelines. For instance, research by Beck et al. in Rwanda showed that an inpatient malnutrition checklist enhanced adherence to WHO protocols, improved communication among staff, and facilitated quality improvement in a low-resourced setting [37].

Findings from the current study contribute new insights by showing that stakeholders at Mwanamugimu recognize their established hospital structures or protocols as effective tools for promoting adherence to prescribed recommendations. Notably, these protocols are implemented systematically and sustainably, which improves long-term compliance among caregivers and staff. The findings of the current study therefore align with previous evidence on the benefits of structured protocols. Like the experiences documented in Rwanda, the results of this study confirm that integrating established hospital protocols into routine practice supports consistent guideline adherence. This reinforces the growing body of evidence that such mechanisms are essential for sustaining high-quality care in low-resource settings.

Investing in structured hospital protocols ensures the systematic, sustainable implementation of evidence-based recommendations and promotes long-term compliance. These protocols enhance consistency, reduce treatment variability, and improve patient outcomes. Policymakers should prioritize their development and integration across community and facility programs by allocating resources for ongoing training and quality improvement, thereby promoting better communication and coordination among healthcare providers.

### Study strengths and limitations

This study involved interviews with caregivers and healthcare providers, enabling triangulation of findings across participant groups and enhancing the richness of the results. Furthermore, the study employed standard methodologies based on COREQ guidelines, facilitating comparisons with similar settings as it reports on comparable attributes. The use of primary data was particularly valuable; ensuring that the information collected was directly relevant to the study’s objectives and tailored to exploring perceptions and experiences of Hospital-Acquired Diarrhea among children with severe acute malnutrition. This approach provided in-depth insights that might not have been achievable using secondary data.

However, the study had certain limitations. Key attributes such as concern, anxiety, financial constraints, emotional impact, extent of health information, and the confidence levels of the care givers were not quantitatively measured as they were beyond the scope of this research. Furthermore, variables, providing deeper insights into risk factors, treatment effectiveness, and patient outcomes would require a quantitative review to enhance evidence-based interventions and improve care strategies.

Our findings suggest that effective management of hospital-acquired diarrhea in children with severe acute malnutrition may be improved by reinforcing health education, timely drug administration, and caregiver engagement. Practical approaches such as structured health talks, routine nutrition assessments, and clearer caregiver-provider communication could enhance adherence and support recovery

## Conclusion

The study highlights an urgent need to strengthen caregiver empowerment through targeted health education, consistent provision of hygiene materials, and structured ward routines. Empowered caregivers demonstrated improved confidence, adherence, and engagement, directly enhancing recovery outcomes for children with severe acute malnutrition. There is need to prioritize integrating psychosocial support, participatory care strategies, and routine health education into hospital nutrition programs. Immediate action is essential to reduce hospital acquired diarrhea, improve treatment effectiveness, and ensure sustainable, high-quality patient care in resource-limited settings. This information will aid in the overall management of severe acute malnutrition in the Uganda and other similar settings.

### Recommendations

Based on our findings, targeted measures may help improve care and reduce hospital-acquired diarrhea in children with children with severe acute malnutrition. Strengthening caregiver empowerment through health education, participatory care strategies, and structured ward routines could enhance confidence, adherence, and engagement, supporting recovery outcomes. Regular evaluation of interventions and clear risk communication within hospitals may help maintain effectiveness and responsiveness. Integrating psychosocial support and supportive reminders for caregivers and providers could further promote timely actions and sustainable improvements. These approaches may enhance the quality and sustainability of hospital nutrition and infection-prevention services in Uganda and similar resource-limited settings.

## Supporting information

S1 File IDI Transcript files. (RAR)

S2 File KII Transcript files. (RAR)

S3 File Code book. (XLSX)

## Abbreviations

AM: Acute Malnutrition
BMI: Body Mass Index
GI: Gastro-intestinal
HAD: Hospital acquired diarrhea
ICP: Infection Control Practices
IDI: In-Depth Interview
IYCF: Infant and Young Child Feeding
KII: Key Informant Interview
LOS: Length of Stay
MAM: Moderate Acute Malnutrition
MIYCAN: Maternal, Infant, Young Child and Adolescent Nutrition
MND: Micro Nutrient Deficiency
MNU: Mwanamugimu Nutritional Unit
MUAC: Mid Upper Arm Circumference
ORT: Oral Rehydration Therapy
RUTF: Ready to Use Therapeutic Feeds
SAM: Severe Acute Malnutrition
UNICEF: United Nations Children’s Fund
WASH: Water, Sanitation and Hygiene
WHO: World Health Organization
WHZ: Weight for Height Z-score.
